# Parenting as a moderator of the neural mechanisms between irritability and inhibition in young children

**DOI:** 10.1016/j.xjmad.2026.100208

**Published:** 2026-10-14

**Authors:** Alexa N. Duran, Victoria Medina, Lea R. Dougherty, Jillian Lee Wiggins

**Affiliations:** a Department of Psychology, San Diego State University, San Diego, CA, 92182, USA; b San Diego State University/University of California San Diego Joint Doctoral Program in Clinical Psychology, USA; c Department of Psychology, University of Maryland, College Park, MD, 20742, USA

**Keywords:** Irritability, Inhibition, Parenting, Neural connectivity, Childhood

## Abstract

**Objective:**

Elevated irritability is a common and impairing feature of early childhood psychopathology and is closely linked to deficits in inhibitory control, alterations in fronto-limbic neural circuitry, and emotional and behavioral difficulties across development. Despite evidence implicating inhibition-related neural mechanisms in irritability, less is known about how environmental factors, particularly parenting behavior, shape these neural associations during this sensitive developmental window. The present study examined whether negative parenting moderates the relationship between irritability symptoms and inhibition-related brain connectivity in school-aged children.

**Method:**

Participants were 182 children aged five to seven years who completed a child-friendly go/no-go (“Whack-a-Mole”) task during functional magnetic resonance imaging (fMRI). We examined whole-brain activation and left/right amygdala functional connectivity across differing levels of irritability symptoms and negative parenting. Neural connectivity contrasts of interest included negative parenting and irritability interaction effects. Positive parenting was controlled in the model.

**Results:**

Results revealed significant negative parenting by irritability interactions for right amygdala connectivity. Children with higher irritability exhibited different patterns of amygdala connectivity during the task as a function of negative parenting, with higher negative parenting associated with less discrimination in connectivity across task demands. Findings were robust to post hoc adjustment for biological sex, race/ethnicity, socioeconomic status, and motion.

**Conclusion:**

These findings highlight negative parenting as a key moderator of neural pathways underlying inhibition and irritability, emphasizing the importance of interventions that reduce vulnerability and support regulatory development in early childhood.

## Highlights

• Negative parenting moderated irritability-related right amygdala connectivity.
• Child-friendly go/no-go fMRI examined inhibitory control in 182 children aged 5–7.
• Moderation was most evident during errors and no-go inhibitory demands.
• Higher negative parenting was linked to less discrimination in task connectivity.

## 1 Introduction

Elevated irritability, characterized by a heightened propensity toward anger and frustration [1], is a core symptom and robust predictor of many developmental and psychiatric disorders in youth [2], [3], [4], [5], [6]. For early school-aged children, a multitude of normative developmental transitions, such as starting school, learning to socialize with peers, and rapidly changing emotional capacities, emerge and may promote frustration from navigating new situations. Although anger and frustration are developmentally normative in early childhood, persistent and dysregulated irritability is associated with significant impairments in emotional and social functioning [7]. Early childhood may therefore represent a critical developmental window for examining regulatory processes associated with irritability before patterns of emotional and behavioral dysregulation become more stable.

One regulatory process that may be relevant to irritability is inhibitory control, or the ability to suppress a dominant or prepotent response when it is inappropriate. Inhibitory control captures one component of regulatory functioning that may be associated with children’s ability to manage frustration, inhibit impulsive responses, and adjust behavior in response to changing task demands. Prior work has linked poorer inhibitory control, behaviorally assessed, and broader executive functioning difficulties with negative emotionality, externalizing problems, and irritability-related outcomes, including a reduced capacity to suppress aggressive or impulsive behaviors [8], [9], [10], [11], [12], [13], [14], [15]. However, associations between behaviorally assessed inhibitory control and irritability are not always strong or consistent, suggesting that inhibitory control should be conceptualized as one candidate regulatory correlate of irritability rather than a direct proxy for emotion regulation.

Neurobiologically, inhibitory control relies in part on prefrontal systems involved in cognitive control and behavioral regulation [16], [17]. Irritability and related forms of emotional dysregulation have also been linked to limbic and fronto-limbic systems, particularly the amygdala and its connectivity with prefrontal regions involved in regulatory control [18], [19], [20], [21], [22]. Although typically studied in emotional contexts, amygdala–prefrontal circuitry may also support responses to inhibitory demands and errors in the absence of emotional stimuli. A non-emotional task may therefore reveal irritability-related differences in regulatory circuitry without confounding effects of emotional content. Yet despite the importance of early childhood, when neural and emotional regulation systems are still maturing [23], task-based functional neuroimaging studies of inhibitory control in early school-aged children remain rare, in part because of methodological challenges associated with scanning very young children. As a result, little is known about how neural systems supporting inhibitory control are associated with irritability during the developmental period when these systems are still consolidating.

Children’s regulatory development also occurs with caregiving environments that may support or strain emerging self-regulatory capacities. Negative parenting has been associated with elevated irritability, poorer self-regulation, and increased risk for emotional and behavioral difficulties [24], [25], [26]. Broadly, negative parenting can include harsh discipline, criticism, rejection, low warmth, or unsupportive responses to children’s distress. Negative parental emotion socialization is a subset of negative parenting that includes punitive, minimizing, or distressed parental responses to children’s negative emotions. From an emotion socialization perspective, these responses may undermine children’s sense of emotional stability and limit opportunities to develop regulatory skills that support inhibition and flexible behavioral control [27], [28], [29]. Consistent with this view, chronic harsh parenting has been linked to disrupted emotion regulation, reactive stress responding, and increased risk for internalizing and externalizing problems [30]. By contrast, positive parenting, broadly characterized by warmth, responsiveness, communication, and support, has been associated with lower irritability and stronger regulatory functioning in children [9], [31], [32], [33]. At the same time, positive parenting may not be uniformly beneficial across all contexts or child profiles, and its associations with child outcomes may depend on children’s individual characteristics and broader family dynamics. These findings support conceptualizing positive and negative parenting as partially distinct dimensions rather than opposite ends of a single continuum. We focused on negative parenting because reducing punitive, minimizing, and distressed responses may address caregiving processes particularly relevant to children’s irritability, whereas positive parenting may represent a distinct protective influence.

Negative parenting may be especially relevant to irritability. Irritability involves heightened anger and frustration, and negative parental emotion socialization captures caregiver responses to children’s negative affective states. Children who frequently express anger or frustration may receive more punitive, minimizing, or distressed responses from caregivers, and caregivers experiencing stress, fatigue, or limited resources may also respond less supportively to children’s negative emotions. Thus, associations between negative parenting and irritability may reflect multiple processes, including parenting effects on children, effects of child behavior/emotions on parenting, shared genetic liability, broader family adversity, and/or caregiver stress. In addition, parent emotion socialization may capture responses that vary across contexts and time rather than a fully stable trait-like exposure. Accordingly, negative parenting in this study is best understood as a contextual correlate and potential moderator of child irritability and regulatory functioning, rather than as a causal exposure.

Prior work suggests negative parenting and child regulatory difficulties may be intertwined across development. For example, punitive parenting behaviors have been linked to irritability trajectories across toddlerhood, particularly among children with lower effortful control [15]. Other developmental models suggest that temperament and parenting may interact in ways consistent with diathesis-stress processes, such that children with greater negative affect or poorer regulation may show heightened vulnerability in less supportive caregiving contexts [34]. Similarly, low effortful control may contribute to harsher parenting and greater irritability jointly predicting later oppositional behavior [35]. Together, these findings suggest that negative parenting may help explain variability in associations between children’s regulatory processes and irritability, while also highlighting the possibility that these associations are bidirectional or shaped by broader family-level factors.

Negative parenting may also be relevant to neural systems implicated in irritability and inhibitory control. Adversity-focused and caregiving models suggest that stressful or unsupportive caregiving environments are associated with differences in prefrontal-limbic circuitry, including amygdala-prefrontal circuitry [18], [36]. Given the amygdala’s established role in irritability, its connectivity with prefrontal control regions may clarify how irritability relates to inhibitory control and whether this association varies with negative parenting. Recent work has identified neural correlates of irritability during inhibitory control tasks [37], but little is known about whether parenting context moderates associations between irritability and neural function during inhibitory control, particularly in early school-aged children.

The present study examined whether negative parenting moderates the association between irritability and amygdala-prefrontal connectivity during go/no-go inhibitory control in school-aged children. Consistent with the broader literature, we use the term “negative parenting” throughout; however, the construct assessed in the current analyses specifically reflects negative parental emotion socialization, or parent-reported unsupportive responses to children’s negative emotions. Based on prior work demonstrating altered amygdala-prefrontal connectivity during inhibitory control in irritable youth [20], we expected moderation effects to emerge most clearly in amygdala connectivity with prefrontal control regions, particularly lateral prefrontal regions such as the middle frontal gyrus. Given that go/no-go tasks also require children to prepare, execute, and inhibit motor responses, we also expected that effects might extend to task-relevant sensorimotor regions, including the precentral and postcentral gyri. We hypothesized that negative parenting, above and beyond positive parenting, would moderate the association between irritability and amygdala–prefrontal connectivity and explored whether this moderation varied across task demands and performance outcomes. Specifically, we expected amygdala–prefrontal connectivity in these children to vary less across task demands and performance outcomes, reflecting differences in how emotional signals are integrated with regulatory processes during challenging regulatory contexts. Reduced differentiation across conditions would suggest less context-sensitive recruitment of amygdala-centered circuitry as inhibitory demands and response outcomes change. Given the cross-sectional and associative design, findings are interpreted as evidence of statistical moderation rather than causal directionality. Understanding how parenting shapes neurobiological pathways underlying emotion regulation has important clinical implications, particularly for parent management interventions targeting irritability and self-regulatory difficulties [4].

## 2 Materials and methods

### 2.1 Participants

227 children were recruited from the San Diego community for the Brains in Growth - Emotions (BIG-Emotions) Study, a longitudinal study enriched for participants with irritable moods and behaviors. Participants were between the ages of five to seven years old (M = 5.61, SD = 0.51) with varying levels of irritability symptoms and completed a child-friendly go/no-go task to elicit neural activity in inhibitory circuits during functional magnetic resonance imaging (fMRI) acquisition. Exclusions were made for those who had a major medical issue that affects the central nervous system, a diagnosis of autism spectrum disorder, and any magnetic resonance imaging (MRI) contraindications (e.g., claustrophobia, metal implants, orthodontic braces). Of the 227 participants, data from individuals were excluded due to a central nervous system medical issue or autism diagnosis (n = 10), incomplete MRI scan or MRI contraindications (n = 26), and unusable MRI data due to motion or scans across multiple days (n = 9). The analytic sample thus included N = 182 participants from the baseline visit of the longitudinal study. Parents of minor participants gave written permission while minors gave verbal assent. Study procedures and consent forms were approved by the San Diego State University Institutional Review Board.

### 2.2 Measures

#### 2.2.1 Parenting

The Children’s Negative Emotions Scale [CCNES] is a 72-item questionnaire that measures how parents respond to their children's negative emotions. Parent-reported items are phrased as statements (e.g., “Tell him/her that's what happens when you're not careful”) and responses are rated on a 7-point Likert scale from 1 (very unlikely) to 7 (very likely). The CCNES measures different dimensions of parent responses including Distress, Punitive, Expressive/Encouragement, Emotion Focused, Problem Focused, and Minimization Reactions. CCNES has demonstrated strong psychometric properties, with subscale internal consistency estimates (Cronbach’s α) typically ranging from.70 to.92, and test-retest reliability coefficients between.62 and.77 over a 4–6 week interval, supporting its reliability and temporal stability [38]. In our sample, the primary caregiver completed the CCNES, which was biological mothers for the large majority of participants (n = 176).

CCNES was divided into two scores, positive and negative parenting, to represent the emotional responses of negative and positive parenting, based on prior literature [20]. Composite scores were created because the subscales corresponding to positive and negative parenting were correlated (Supplement; Table S1). A composite for negative parenting score was created by averaging the total scores of Distress, Punitive, and Minimization Reactions subscales of the CCNES, which demonstrated strong internal consistency (Cronbach’s α =.88). Similarly, positive parenting was calculated by averaging the total scores of the Expressive/Encouragement, Emotion Focused, and Problem Focused Reactions subscales, and demonstrated exceptional internal consistency (Cronbach’s α =.92). Mean imputation was conducted for six participants who selected “Prefer not to respond” for two out of the thirty-six questions used to create the positive parenting composite score.

#### 2.2.2 Irritability symptoms

Parents reported on their children’s irritability using the Multidimensional Assessment Profiles – Temper Loss (MAPS-TL) [39], a 22-item questionnaire designed to assess the severity of irritability along a dimensional spectrum, capturing key developmental factors that differentiate typical early childhood misbehavior from clinically significant disruptive behavior [40]. Items are statements (e.g., “Lose temper or have a tantrum ‘out of the blue’ or for no reason”) ranked on a 6-point Likert scale from 0 (never) to 5 (many times a day). In a diverse sample of 1488 preschoolers, the MAPS Temper Loss scale demonstrated excellent reliability, with a Cronbach’s alpha of.97 and a 1-week test-retest intra-class correlation coefficient (ICC) of.94 [39]. MAPS-TL also demonstrates excellent internal consistency (Cronbach’s α =.97) within our sample.

#### 2.2.3 fMRI data acquisition

Participants were scanned using a 3 Tesla Siemens Magnetom Prisma with a 30-channel head coil. Multiband procedures were utilized to increase spatial and temporal resolution. T2 blood oxygen level dependent (BOLD) images were acquired using a 3D multiband EPI pulse sequence across 3 runs. For whole-brain coverage, there were 60 interleaved sagittal slices approximately parallel to the AC-PC line (voxel size = 2.4 ×2.4 ×2.4 mm, 316 image volumes per run, matrix size = 90 ×90 ×60, acceleration factor=6, TR=800 ms, TE=30.8, flip angle=52°, FOV=216 mm). Anatomical images with prospective motion correction (T2-weighted MPRAGE PROMO) were obtained for anatomical localization and spatial normalization (176 sagittal slices, flip angle=9°, matrix size = 256 ×256 ×176, FOV=256 mm, voxel size = 1 ×1 ×1 mm).

#### 2.2.4 Inhibition task

During fMRI acquisition, participants completed the Whack-a-Mole Task, a validated, child-friendly go/no-go paradigm designed to assess neural connectivity associated with response inhibition [41], [42]. Before scanning, children were familiarized with the task stimuli and completed practice trials to confirm that they understood which stimuli required a button press and which required no response. During the task, participants were instructed to press a button as quickly as they can in response to frequent “go” stimuli (a cartoon mole or chipmunk) and to withhold their response to infrequent “no-go” stimuli (eggplants) (Figure S1). Only one character was presented per trial. The task was designed to manipulate inhibitory demands by varying the number of preceding go trials (ranging from 1 to 7), thereby increasing the prepotency of the go response and enhancing the cognitive control required on subsequent no-go trials. Four trial types were examined: correct go (prepotent responses), incorrect go (attention lapse), correct no-go (successful inhibition), and incorrect no-go (failed inhibition). Go versus no-go represented trial type, whereas correct versus incorrect represented participant performance. Each individual trial began with a go or no-go image displayed for 1000 ms, followed by a jittered interstimulus interval averaging 1500 ms, with intervals ranging from 500 to 3500 ms allowing for appropriate temporal resolution of the BOLD response. During scanning, participants completed three runs of the task, with each run including two 45-trial blocks composed of 80% Go trials. Across each run, this yielded 72 Go trials and 18 No-Go trials. Between runs, the experimenter provided performance-based reminders. Children were encouraged to respond as quickly as they could when Go accuracy was below 90%, mean Go reaction time exceeded 800 ms, or No-Go accuracy exceeded 50%. Children were encouraged to slow down when No-Go accuracy fell below 20%. These adjustments were intended to maintain sufficient variability in task performance and ensure an adequate number of correct and incorrect trials for analysis. No-go trials preceded by longer go sequences were more common in the first and last runs to enhance inhibitory load. Stimuli were presented via a projection system and viewed through a mirror mounted on the head coil.

### 2.3 Analyses

#### 2.3.1 fMRI data preprocessing and first-level analysis

Analysis of Functional NeuroImages (AFNI; https://afni.nimh.nih.gov/afni) was utilized for preprocessing protocols, which included functional image realignment, slice-time correction, spatial smoothing of 4 mm FWHM, and non-linear registration for spatial standardization to the Talairach template [43]. Post-processed functional images were resampled to 2-mm voxels. Image volume pairs with Euclidean norm motion derivative > 0.30 mm were censored from individual-level analysis. All participants evidenced mean Euclidean norm motion derivative (head motion) ≤ 0.30 mm.

Individual-level brain activations for the four tasks, correct Go, incorrect Go, correct No-Go, and incorrect No-Go, were modeled using an event-related design. The regressors were generated by modeling the onset of target/distractor presentation convolved with a gamma hemodynamic function (GAM). Voxel-wise connectivity images were created for all go/no-go periods by calculating amygdala connectivity (left and right separately) within the brain. The amygdala was selected based on its established involvement in irritability [21], [22], [37], [44], [45], [46], [47], [48], [49], [50] and prior research linking irritability, parenting, and inhibitory processes [24], [37], [51], [52], [53]. The seed region was defined based on the Talairach atlas in AFNI (left amygdala = 1288 mm^3^; right amygdala = 1280 mm^3^).

#### 2.3.2 Second level analysis

Since the primary analyses focused on task-dependent amygdala connectivity, behavioral performance and task-evoked activation analyses were conducted to characterize whole-brain neural engagement and aid interpretation of the connectivity findings. Behavioral performance on the Go/No-Go task was examined to characterize task engagement and inhibitory-control performance in this developmental sample. Behavioral indices included accuracy for Go and No-Go trials and reaction time for Go trials. We examined whether irritability symptoms and negative parenting were associated with behavioral performance.

Group-level activation analyses were performed using AFNI’s 3dLMEr [54]. Task condition (Go, No-Go) and response accuracy (correct, incorrect) were modeled as within-subject factors, with irritability and negative parenting as between-subject variables and positive parenting as a covariate. To evaluate whether this covariate influenced the findings, a model excluding positive parenting is reported in the Supplement (Figure S2; Table S4). Whole-brain activation analyses were conducted to contextualize the connectivity findings and focused on interactions between negative parenting and irritability.

Connectivity models examined task-dependent amygdala connectivity and were run separately for the left and right amygdala seeds. All interaction effects were estimated within a single omnibus model. The Negative Parenting × Irritability × Condition × Accuracy interaction was the primary contrast and was characterized using condition- and accuracy-specific contrasts from the same model; lower-order interactions were interpreted as secondary and in the context of the four-way effect. For both activation and connectivity analyses, statistical maps used a voxel-wise threshold of p < .001. Cluster-size thresholds were determined using AFNI’s 3dClustSim with mixed-model spatial autocorrelation (-acf) and NN1 bi-sided clustering, controlling family-wise error at α = .01. The simulations used the same whole-brain mask, 2-mm voxel resolution, and clustering parameters as the group analyses, yielding a threshold of k ≥ 39 voxels (≥312 mm³). The relatively small threshold may reflect the high spatial and temporal resolution afforded by multiband imaging, together with a favorable signal-to-noise ratio. Given the small cluster-size threshold, voxel-wise FDR correction was additionally applied using AFNI’s 3dFDR (an adaptation of the Benjamini-Hochberg method) as a robustness check, with significance defined as *q* < .05. Only findings that survived both correction procedures were interpreted.

#### 2.3.3 Covariates

Child sex, race/ethnicity, household income, primary caregiver education, and average censored motion were included as covariates in post hoc analyses to account for potential influences on study results. These variables were not the focus of the primary hypotheses but were examined to evaluate the robustness of observed effects when adjusting for these factors.

Child sex was coded as 0 = male and 1 = female. Race/ethnicity was treated as a categorical variable and dummy coded, with White as the reference group in post hoc analyses because they comprised the numerical majority. Household income and primary caregiver education were included as indicators of socioeconomic status. Primary caregivers selected the category that best represented their household income before taxes from the following options: less than $20,000; $20,000–$39,999; $40,000–$59,999; $60,000–$79,999; $80,000–$99,999; $100,000–$119,999; $120,000–$250,000; and more than $250,000. Similarly, both primary and coparent caregivers reported their highest level of education from the following options: 8th grade or less; some high school; some college (or 2-year degree); 4-year college degree; master’s degree; and doctoral degree. Only primary caregiver education was included in analyses due to a moderate positive correlation between caregivers’ education levels, r_s_(167) = .51, p < .001.

Values were extracted and averaged for each significant connectivity cluster and exported to RStudio v.2024.04.1 [55]. For each cluster, the analyses were recreated with the additional factor added to the model as a covariate to determine whether the cluster was still significant after adjusting for the additional covariates. Packages to conduct the analyses included lme4 [56], lmertest [57], matrix [58], psych [59] and car [60].

## 3 Results

The sociodemographic information of the analytic sample is presented in Table 1. Spearman correlations among the variables of interest and covariates are reported in Table 2.

**Table 1:** Sample Characteristics.

	**Mean**		**Standard Deviation**		**Range**		**Skewness Statistic**		**Kurtosis Statistic**	
Age (years)	5.6		0.5		5–7		0.16		-1.4	
CCNES										
Negative Parenting Score	79.0		22.1		43–174		1.34		2.62	
Positive Parenting Score	81.2		24.8		41–188.8		-1.04		1.72	
MAP-TL										
Irritability Symptoms	29.8		18.75		0–91		0.91		0.81	
SES score*	-0.01		0.87		(-2.33)-1.57		-0.56		-0.26	
		**N**		**Percentage**						
Gender										
Female		97		53.3%						
Male		84		46.2%						
Transgender Female		1		0.5%						
Race/Ethnicity										
African American/Black		8		4.4%						
Asian		6		3.3%						
Native American		1		0.5%						
Native Hawaiian/Pacific Islander		1		0.5%						
White		110		60.4%						
Multiracial		48		26.4%						
Other/Unknown		8		4.4%						
Hispanic Origin										
Yes		58		31.9%						
No		120		66.0%						
Don’t know/Prefer not to say		4		2.5%						

*Note.* CCNES = Coping with Children’s Negative Emotions Scale; MAP-TL = Multidimensional Assessment Profiles –Temper Loss; SES = Socioeconomic Status; * z-scored. Summary of participant demographics, including age, gender, and other relevant characteristics (N = 182). CCNES Negative Parenting score consists of the participant’s average score across Distress, Punitive, and Minimization Reactions subscales total score. CCNES Positive Parenting score consists of the participant’s average score across Expressive/Encouragement, Emotion Focused, and Problem Focused Reactions subscale total scores. SES score consists of the means of standardized household income and parental education (primary parent and co-parent).

**Table 2:** Spearman Correlation Matrix for Predictor Variables and Covariates.

	1	2	3	4	5	6	7
1. Negative Parenting	-						
2. Positive Parenting	-0.16*	-					
3. Irritability	0.03	-0.12	-				
4. Sex (Female)	0.03	0.11	-0.16*	-			
5. Household Income	-0.02	-0.23^***^	0.12	0.02	-		
6. Primary Caregiver Education	-0.20*	-0.09	0.04	0.03	0.39^***^	-	
7. Average Censored Motion	-0.01	0.05	0.08	-0.05	-0.08	-0.17*	-

*Note.* * p < .05; ^**^ p < .01; ^***^ p < .001

Based on behavioral results, irritability was not associated with the reaction time for Go (r(180) = 0.01, *p* = .93), accuracy of Go (r(180) = -0.02, *p* = .84), or No-Go trials (r(180) = 0.05, *p* = .49). The average Go reaction time was 643.69 ms (SD = 83.83). Neither negative nor positive parenting was behaviorally associated with reaction time for Go (r(180) = -0.02, *p* = .80; r(180) = 0.05, *p* = .50), accuracy of Go (r(180) = 0.01, *p* = .88; r(180) = -0.05, *p* = .51), or No-Go trials (r(180) = 0, *p* = .97; r(180) = 0.04, *p* = .57), respectively. The average accuracy was 0.93 (SD = 0.08) for Go trials and 0.67 (SD = 0.16) for No-Go trials. For neuroimaging results, only contrasts of interest are reported below; the full results are available in the Supplement (Table S2 and S3). Although some significant clusters were located in cerebellar and brain stem regions, these clusters are not further discussed due to a lack of optimization for these regions in the scanning protocol. No significant results emerged for neural activation in contrasts of interest and no significant results emerged in the left amygdala seed; therefore, only right amygdala seed connectivity results are reported. The pattern of findings was comparable, if not stronger, in a model excluding positive parenting (Supplement; Figure S2; Table S4).

### 3.1 Negative parenting x irritability x condition x accuracy

We observed significant Negative Parenting x Irritability x Condition x Accuracy interactions for multiple regions including right amygdala connectivity with middle frontal, postcentral, precentral, and temporal gyri; hippocampus; and precuneus/cuneus regions (Table 3, Fig. 1A). The interactions were similar across all clusters and primarily driven by irritability-related differences between the go vs. no-go conditions in incorrect trials, when participants had lower negative parenting. By contrast, there were few irritability-related connectivity differences in the go vs. no go conditions in correct trials, regardless of negative parenting level, and incorrect trials when there was higher negative parenting.

**Table 3:** Model Results from Connectivity Analysis.

**Right Amygdala Seed**					
***Negative Parenting x Irritability***					
**k**	**χ**^**2**^**(2)**	**x**	**y**	**z**	**Region**
606	44.4	-49	-13	44	Postcentral & precentral gyri, rolandic operculum
513	48.2	5	-69	34	Precuneus
498	36.6	45	-41	34	Inferior/superior parietal lobule, supramarginal & angular gyri
276	46.1	53	-3	34	Precentral & postcentral gyri
180	30.3	49	1	42	Precentral & middle frontal gyri
174	34.7	-1	-35	32	Middle/posterior cingulate cortex
168	46.3	-37	-55	22	Middle temporal & angular gyri
162	26.4	37	57	22	Middle frontal & middle orbital gyri
116	32.6	39	-61	10	Middle temporal & middle occipital gyri
110	33.1	51	23	28	Middle frontal & inferior frontal (p. triangularis) gyri
82	25.6	53	-41	4	Middle temporal & superior temporal gyri
82	33.2	-45	-55	42	Inferior parietal lobule, angular gyrus
69	44.4	55	-29	22	Superior temporal & supramarginal gyri, rolandic operculum
64	34.9	57	7	8	Temporal pole, rolandic operculum, inferior frontal gyrus (p. opercularis)
61	43.7	61	-45	26	Supramarginal & superior temporal gyri
57	30.2	55	-7	16	Rolandic operculum, postcentral gyrus
47	28.9	47	11	16	Inferior frontal gyrus (p. opercularis) & superior medial gyri
44	31.2	3	31	36	Middle cingulate cortex
42	26.0	45	9	32	Inferior frontal gyrus (p. opercularis)
***Negative Parenting x Irritability x Condition***					
**k**	**χ**^**2**^**(2)**	**x**	**y**	**z**	**Region**
216	42.7	51	-5	32	Postcentral & precentral gyri
106	41.3	-43	-13	28	Postcentral & precentral gyri
58	31.2	-45	-13	42	Postcentral gyrus
48	32.8	-41	-23	-10	Middle temporal & postcentral gyri
47	20.4	59	-11	18	Rolandic operculum, superior temporal gyrus
40	28.8	41	-61	52	Superior/inferior parietal lobule, angular gyrus
***Negative Parenting x Irritability x Accuracy***					
**k**	**χ**^**2**^**(2)**	**x**	**y**	**z**	**Region**
865	50.8	-49	-13	42	Postcentral & precentral gyri, rolandic operculum
629	52.0	47	-5	30	Precentral & postcentral gyri
391	38.0	45	-41	34	Inferior/superior parietal lobule, supramarginal gyrus
341	35.6	5	-71	34	Precuneus, cuneus
302	45.9	57	9	6	Inferior frontal gyrus (p. opercularis/p. triangularis), rolandic operculum, insula lobe
206	46.2	7	-73	8	Calcarine & lingual gyri
170	40.3	1	-29	26	Posterior/middle cingulate cortex
158	44.9	-37	-55	22	Middle temporal & angular gyri
144	27.4	37	41	10	Middle frontal, middle orbital, & inferior frontal (p. Orbitalis) gyri
135	28.9	-23	-7	4	Insula lobe, putamen
129	32.0	27	-9	4	Putamen, insula lobe
118	30.7	63	-45	8	Middle temporal & superior temporal gyri
118	34.0	51	23	26	Inferior frontal (p. triangularis) & middle frontal gyri
107	29.0	63	-5	18	Rolandic operculum, postcentral gyrus
85	31.9	11	-19	4	Thalamus
85	37.4	37	-63	12	Middle temporal & middle occipital gyri
82	39.0	5	-27	52	Middle cingulate cortex, SMA
76	44.8	55	-29	22	Superior temporal & supramarginal gyri, rolandic operculum
70	30.9	-11	-67	24	Precuneus, cuneus, superior occipital gyrus
67	43.6	61	-45	26	Supramarginal & superior temporal gyri
66	28.6	-49	-41	38	Inferior parietal lobule
58	31.7	-47	-65	14	Middle temporal, angular & middle occipital gyri
56	25.7	-29	57	-6	Middle orbital gyrus
51	25.9	1	-3	54	SMA, middle cingulate cortex
50	21.4	5	-67	-2	Lingual gyrus
45	27.2	39	-9	54	Middle/superior frontal & precentral gyri
44	24.8	5	65	-6	Mid orbital & rectal gyri
***Negative Parenting x Irritability x Condition x Accuracy***					
**k**	**χ**^**2**^**(2)**	**x**	**y**	**z**	**Region**
197	40.5	51	-5	32	Precentral & postcentral gyri
92	38.6	-43	-13	28	Postcentral & precentral gyri
74	38.2	7	-71	34	Precuneus, cuneus
64	32.7	-17	39	18	Middle frontal gyrus
61	36.2	-41	23	-10	Hippocampus, middle temporal gyrus
43	26.7	-49	13	44	Postcentral & precentral gyri

Significant cluster results show the associations between negative parenting scores, irritability symptoms, and neural connectivity during go/no-go conditions controlling for positive parenting in the Whack-a-Mole task.

**Fig. 1:**
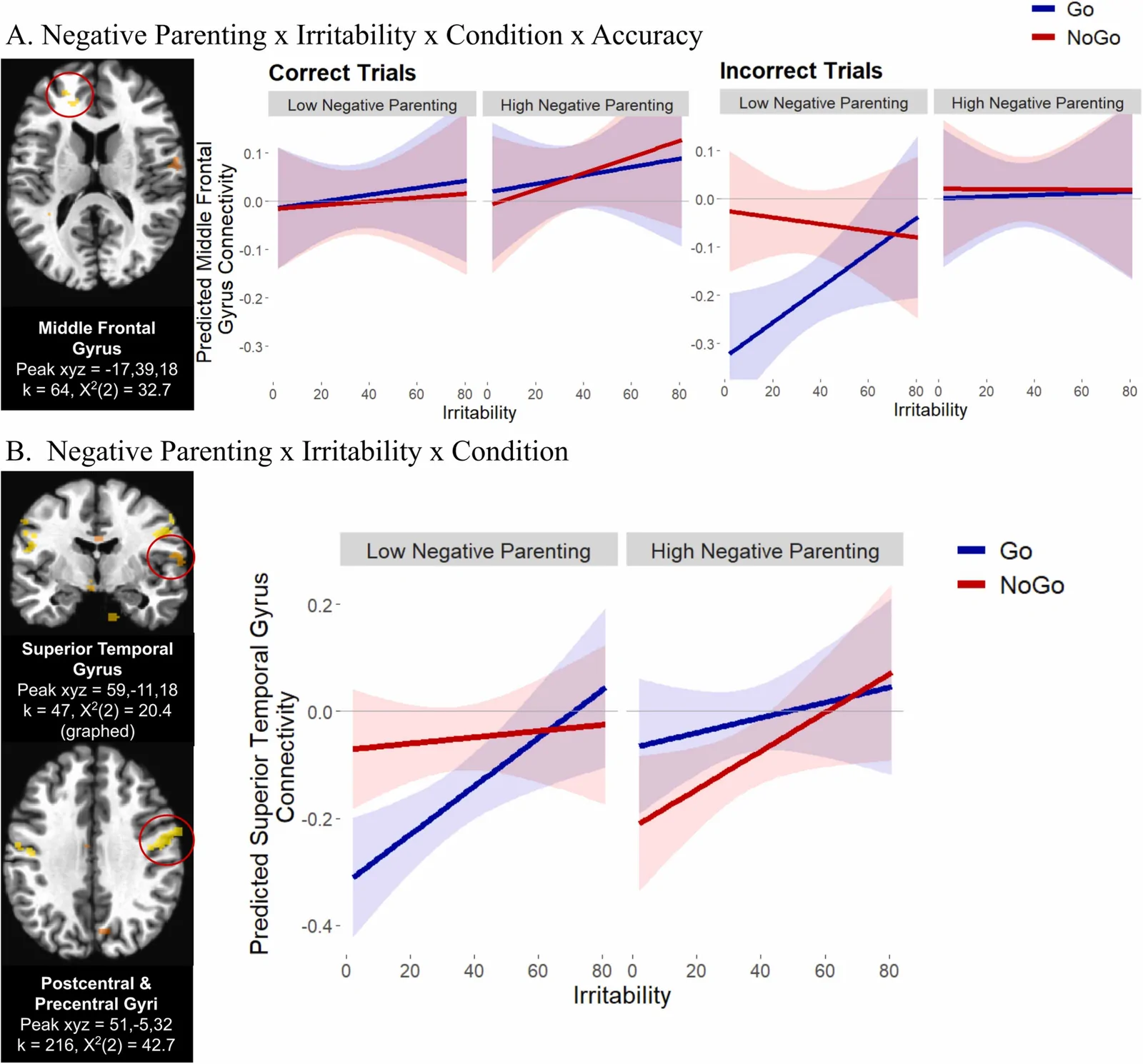
The moderating effects of negative parenting on irritability and inhibition-related whole-brain connectivity based on right amygdala seed region and controlling for positive parenting. For all figures: For illustrative purposes, all graphs represent predicted connectivity values for indicated clusters based on the high and low negative parenting and irritability scores (high and low were defined as ± 1 standard deviation from the mean). Shaded regions represent ±1 standard error of the model-predicted amygdala connectivity. Selected significant clusters are shown using a voxel-wise threshold of p < .001 and a cluster-level family-wise error threshold of α = .01. When a contrast comprises several regions with similar patterns, only one cluster is presented as an example.

### 3.2 Negative parenting x irritability x condition

We observed significant Negative Parenting x Irritability x Condition interactions for multiple regions including right amygdala connectivity with precentral, postcentral, temporal, parietal, and angular gyri; superior/inferior parietal lobule; and rolandic operculum regions (Table 3, Fig. 1.B). Overall, irritability-related changes in go vs. no-go connectivity show distinct patterns. Under lower levels of negative parenting, increases in amygdala connectivity were steeper for go vs. no-go conditions, however, under higher levels of negative parenting, irritability-related connectivity increases were steeper for no-go vs. go.

### 3.3 Negative parenting x irritability x accuracy

We observed significant Negative Parenting x Irritability x Accuracy interactions for multiple regions including right amygdala connectivity with frontal, precentral, postcentral, supramarginal, temporal, occipital, parietal, rectal, calcarine, lingual gyri; posterior/middle cingulate cortex; putamen; thalamus; insula lobe; supplementary motor area; rolandic operculum; and precuneus/cuneus regions (Table 3, Fig. 2.A). Across all clusters, the pattern of the interaction was similar and primarily driven by negative parenting-related differences between correct vs. incorrect trials. Under lower levels of negative parenting, amygdala connectivity displayed a gradual decrease with greater irritability. By contrast, under higher levels of negative parenting, connectivity increased as irritability scores increased, with a steeper increase for incorrect trials. Therefore, irritability-related changes in amygdala connectivity and accuracy-related differences varied as a function of negative parenting level.

**Fig. 2:**
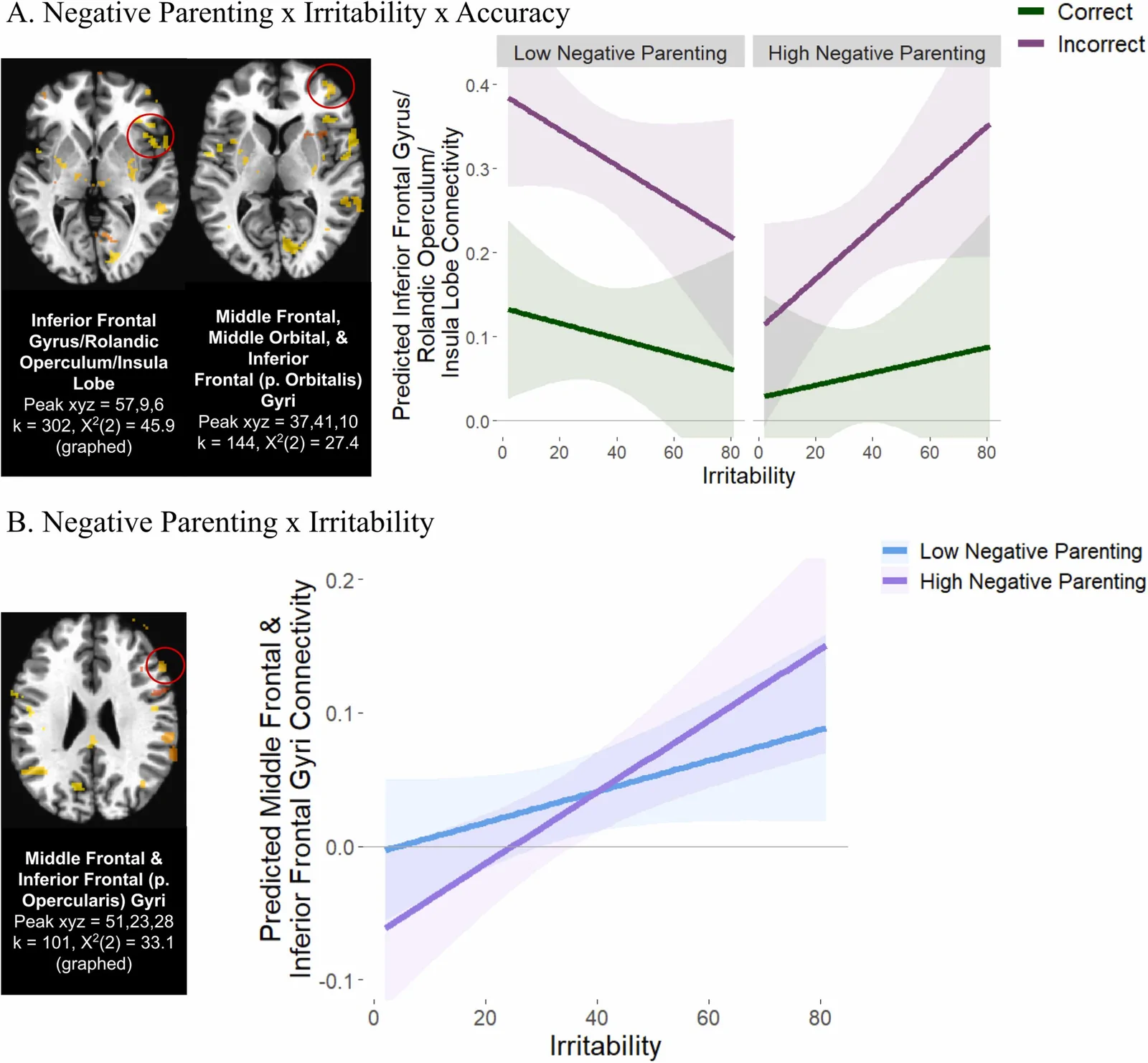
The moderating effects of negative parenting on irritability and inhibition-related whole-brain connectivity based on right amygdala seed region and controlling for positive parenting.

### 3.4 Negative parenting x irritability

We observed significant Negative Parenting x Irritability interactions for multiple regions, including right amygdala connectivity with frontal, precentral, postcentral, supramarginal, temporal, occipital, parietal, rectal gyri; posterior/middle cingulate cortex; supplementary motor area; rolandic operculum; and precuneus/cuneus regions (Table 3, Fig. 2.B). Across all clusters, the amygdala connectivity showed steeper irritability-related increases when there was higher negative parenting.

### 3.5 Additional analyses

Post hoc analyses of biological sex, race/ethnicity, socioeconomic status and average censored motion indicated that the results were not primarily driven by these additional factors after accounting for them separately (see Supplement; Table S5).

## 4 Discussion

In recent years, irritability has garnered interest as a top problem facing pediatric clinicians, motivating increasing research into neurodevelopmentally mapping the mechanisms of irritability, including emerging results on inhibition. Despite this burgeoning evidence base for inhibition’s link to irritability, the questions of “for whom,” “when,” and “how” inhibition may be associated with irritability have lagged. Parenting has long been implicated as a key environmental context for emotion regulation, but little is known about how parenting behaviors are associated with the neural organization of inhibitory control in irritable children. Here, we deployed a neuroimaging task to examine whether parenting context moderates inhibitory-related neural correlates of irritability. Moreover, we did so in a crucial period of rapid neurodevelopmental change, early school age, when timely intervention may help prevent downstream effects on functional outcomes (e.g., Dougherty et al. [3]). By examining irritability-related patterns of amygdala connectivity in one of the youngest task-based fMRI samples studied to date, this work addresses a critical gap in understanding early neural organization of emotion and cognitive control in a highly plastic developmental period relevant for intervention.

To contextualize the connectivity findings, we first examined task performance and task-evoked neural activation. Behaviorally, irritability and negative parenting were not significantly associated with Go/No-Go performance indices, suggesting that the observed neural moderation effects were not accompanied by detectable differences in overt task performance. Since performance-based reminders were used to maintain comparable performance and sufficient correct and incorrect trials, restricted behavioral variability may have limited our ability to detect behavioral associations. The connectivity findings may identify neural differences occurring despite similar task performance; however, without corresponding behavioral effects, they cannot be interpreted as evidence of impaired inhibitory control, emotion regulation, or error processing. Whole-brain neural activation analyses also did not reveal significant associations with irritability and negative parenting interactions. Thus, the findings are specific to task-dependent amygdala connectivity and should not be generalized to behavioral inhibition or regional activation. Together, the behavioral and activation results provide important context: children appeared able to perform the task, but irritability and parenting were most evident in connectivity patterns rather than in observable performance or whole-brain activation.

The primary finding was the four-way interaction among negative parenting, irritability, task condition, and response accuracy, indicating that moderation of irritability-related amygdala connectivity varied across inhibitory demands and performance outcomes. Secondary analyses characterized this interaction within specific condition and accuracy combinations; lower-order patterns were therefore interpreted as secondary components of the higher-order effect rather than as independent findings. Most relevant to our hypothesis, the four-way interaction involved right amygdala connectivity with the middle frontal gyrus, a prefrontal region implicated in cognitive control. Effects also involved precentral and postcentral regions, consistent with the motor-response demands of the go/no-go task. Other regional findings are interpreted cautiously given their broader distribution.

Decomposition of the four-way interaction indicated that negative parenting moderated irritability-related amygdala connectivity most clearly during incorrect trials, with the pattern differing between go and no-go conditions. In contrast, correct trials showed more similar irritability-related patterns across levels of negative parenting. During correct responses, amygdala connectivity tended to increase across irritability for both go and no-go conditions regardless of negative parenting levels, suggesting that negative parenting was less relevant to irritability-related neural connectivity during successful task performance. In contrast, negative parenting was more strongly implicated in irritability-related neural connectivity during incorrect responses, including failed inhibition and attention lapses. This pattern indicates that the observed connectivity associations were most apparent during incorrect trials, although the absence of behavioral associations limits conclusions about error processing or emotional salience. One possibility is that errors elicit frustration, self-monitoring, or concern about negative evaluation, to which children exposed to greater negative parenting may be especially sensitive; however, these processes were not measured in the present study. Although the present task cannot determine whether children were frustrated with themselves, anticipating caregiver consequences, or engaging broader error-processing systems, the concentration of effects during incorrect trials suggest that future work should directly assess affective responses to errors and examine error-processing regions alongside amygdala connectivity.

Within the four-way interaction, the moderating role of negative parenting also differed between go and no-go trials. Specifically, lower negative parenting was associated with greater irritability-related increases during go trials, whereas higher negative parenting was associated with greater increases during no-go trials. Because this pattern was embedded within higher-order task condition and accuracy effects, it should be interpreted cautiously as evidence that parenting context may alter when irritability-related amygdala connectivity becomes most pronounced during the task. One interpretation is that, under higher levels of negative parenting, irritability-related neural differences may be more apparent when children are expected to inhibit a prepotent response, whereas under lower levels of negative parenting, irritability-related connectivity may be more apparent during response execution. This pattern may have implications for intervention; coaching caregivers to respond supportively during high-demand situations, such as when children are asked to stop a preferred activity, wait quietly, or recover from mistakes, may be especially relevant for children with elevated irritability. However, given the cross-sectional design, these implications should be viewed as hypotheses for future longitudinal and intervention research rather than as evidence that parenting caused the observed neural patterns.

Although positive parenting was not examined as a moderator, it remains a potentially important protective factor. Positive and negative parenting are interrelated but distinct dimensions; therefore, supportive parenting may not simply offset the potential effects of punitive, minimizing, or distressed responses. We focused on negative parenting based on our primary hypothesis and included positive parenting as a covariate to estimate the unique moderating role of negative parenting while limiting additional whole-brain analyses. Parent-focused interventions nevertheless often emphasize increasing warmth, responsiveness, and positive reinforcement alongside reducing harsh, punitive, or minimizing responses. Thus, the findings indicate that negative parenting explains unique variability in irritability-related amygdala connectivity after accounting for positive parenting. Future work should examine positive parenting independently and as a moderator to determine whether it buffers irritability-related neural vulnerability. Irritability that remains elevated in supportive caregiving contexts may also reflect child-specific, genetic, or other environmental influences that require different intervention targets.

Overall, the four-way interaction suggests that the association between irritability and amygdala connectivity varies jointly according to parenting context, inhibitory demand, and response accuracy. Negative parenting appeared most relevant during incorrect responses and situations requiring inhibition, although these condition-specific patterns require replication. This distinction has important implications for intervention because it suggests that inhibitory-control components of intervention may need to consider the child’s caregiving context, particularly parenting responses in different situations. Here, negative parenting appeared most relevant for irritability-related connectivity in some situations, such as making errors or being expected to inhibit a response, rather than others. For children exposed to higher levels of negative parenting, irritability interventions may need to address how caregivers respond during these high-demand or error-prone moments. At the same time, these results should be interpreted within a bidirectional developmental framework. Children with higher irritability or poorer inhibitory control may evoke more negative or distressed parenting responses, and caregiver regulatory capacities may contribute both to parenting behaviors and to children’s neurocognitive functioning through shared genetic or environmental influences. Taken together, these results highlight that irritability may reflect multiple developmental pathways associated with parenting context, child characteristics, and broader family-level processes.

### 4.1 Limitations

There are several limitations to consider. First, this study examined a single time point and does not consider longitudinal patterns. We cannot make causal inferences. A longitudinal design would allow for stronger conclusions about whether neural patterns precede, follow, or co-develop with differing environmental factors, such as negative parenting. Relatedly, the observed associations may reflect bidirectional processes, including child-driven effects on parenting, caregiver stress or fatigue, shared genetic influences, or broader family adversity. Second, while the Whack-a-Mole Go/No-Go task is well-validated and developmentally suitable, performing the task within an MRI scanner introduces a setting distinct from everyday environments and may reduce ecological validity. Such artificial conditions might impact children’s natural responses, raising concerns about the generalizability of our findings to real-world contexts. To mitigate this, considerable effort was made to familiarize children with the scanning environment, and we employed a highly engaging, child-friendly task designed specifically for pediatric populations. Third, behavioral performance and whole-brain activation analyses did not show significant associations with irritability or negative parenting, indicating that the present findings are specific to amygdala connectivity and should not be generalized to overt inhibitory-control performance or regional activation without further replication. Fourth, although the amygdala was selected based on its established involvement in irritability, its use as the sole seed limits conclusions about connectivity within broader cognitive-control networks. Future studies should include prefrontal or frontoparietal seeds to determine whether these findings extend beyond amygdala-centered circuitry. Finally, negative parenting was operationalized using the CCNES, which focuses on negative parental emotion socialization and does not capture all dimensions of negative parenting. Future studies should also examine positive parenting more directly, given its clinical relevance and its potential to buffer risk associated with irritability and negative caregiving contexts.

### 4.2 Conclusions

The results of the present study extend existing research linking negative parenting to irritability and inhibitory control by demonstrating that parenting context moderates how irritability is reflected in amygdala-centered neural organization during inhibition, focusing on an early developmental period, early school age, when plasticity is high and timely interventions to prevent downstream psychopathology may be especially important. Such functional MRI insights have thus far eluded researchers due to the high methodological challenges with scanning early school-age irritable children. Here, using developmentally optimized protocols to maximize compliance and data quality in this young sample, our observed effects highlight differences in the organization and discrimination of amygdala connectivity across task conditions and performance outcomes as a function of negative parenting. These effects occurred without corresponding behavioral or activation differences, although their functional significance requires further investigation. These findings underscore the importance of considering parenting behaviors as salient contextual moderators when interpreting neural correlates of irritability and inhibition in early childhood.

The results are conceptually relevant to parent-focused interventions that aim to modify how caregivers respond to children’s negative emotions. In particular, the results suggest that parenting behaviors may be associated with how emotional signals are integrated with control-related neural systems during challenging situations, especially when children make mistakes or must inhibit a prepotent response. Future research should consider incorporating cultural measures beyond race/ethnicity and socioeconomic status used in this study and measures that characterize parenting styles to accurately capture potential differences in parenting that may influence regulatory trajectories and differentiate them from irritability-related outcomes. Additionally, parenting research could utilize a variety of measures capturing harsh discipline, criticism, rejection, warmth, responsiveness, and positive reinforcement to more fully capture the range of caregiving behaviors and identify which aspects are most relevant for parent-focused interventions.

## Ethics declaration

Written informed consent to take part in the study and to publish the article has been obtained from all participants or their legal representatives. The privacy rights of participants have been observed.

This study was performed in compliance with relevant laws, regulatory frameworks and guidelines where the research took place. This study was approved by the San Diego State University Institutional Review Board. (Approval No. HS-2021–0003)

## Funding

This work was supported by the 10.13039/100000002National Institutes of Health [grant R01 MH121385].

## Declaration of Competing Interest

The authors declare that they have no known competing financial interests or personal relationships that could have appeared to influence the work reported in this paper.
