# Supplementary material for: Parenting as a moderator of the neural mechanisms between irritability and inhibition in young children
Source: J Mood Anxiety Disord. 2026 Oct 14;16:100208. doi: 10.1016/j.xjmad.2026.100208 (PMC13696339; doi:10.1016/j.xjmad.2026.100208)
Supplement: Supplementary file 1 [file mmc1.docx]

**Supplemental Materials**

**Figure S1. Whack-a-Mole Inhibition Task Paradigm**
Figure S2. The moderating effects of negative parenting on irritability and inhibition-related whole-brain connectivity based on right amygdala seed region. For all figures: For illustrative purposes, all graphs represent predicted connectivity values for indicated clusters based on the high and low negative parenting and irritability scores (high and low were defined as ± 1 standard deviation from the mean). Shaded regions represent ±1 standard error of the model-predicted amygdala connectivity. Selected significant clusters are shown using a voxel-wise threshold of p < .001 and a cluster-level family-wise error threshold of α = .01. When a contrast comprises several regions with similar patterns, only one cluster is presented as an example.

Figure S3. The moderating effects of negative parenting on irritability and inhibition-related whole-brain connectivity based on right amygdala seed region.

Table S1. Pearson Correlations Between Subscales of the Coping with Children’s Negative Emotions Scale (CCNES)

|  | 1 | | 2 | | 3 | | 4 | | 5 | | 6 |
| --- | --- | --- | --- | --- | --- | --- | --- | --- | --- | --- | --- |
|  |  | |  | |  | |  | |  | |  |
| 1. Distress Reactions | - | |  | |  | |  | |  | |  |
| 2. Punitive Reactions | -0.52^***^ | | - | |  | |  | |  | |  |
| 3. Minimization Reactions | 0.45^***^ | | 0.68^***^ | | - | |  | |  | |  |
| 4. Emotion Focused Reactions | -0.14 | | 0.00 | | 0.17^*^ | | - | |  | |  |
| 5. Problem Focused Reactions | -0.30^***^ | | -0.30^***^ | | -0.16^*^ | | 0.56^***^ | | - | |  |
| 6. Expressive Encouragement | -0.37^***^ | | -0.42^***^ | | -0.34^***^ | | 0.30^***^ | | 0.62^***^ | | - |
|  |  |  | |  | |  | |  | |  | |

*Note.* ^*^ p<.05; ^**^ p<.01; ^***^ p<.001

**Table S2. Model Results from Connectivity Analysis**

| **Right Amygdala Seed** | | | | | | | | |
| --- | --- | --- | --- | --- | --- | --- | --- | --- |
| ***Negative Parenting Main Effect*** | | | | | | | | |
| **k** | **χ^2^(2)** | | **x** | **y** | | **z** | **Region** | |
| 63 | 21.9 | | 47 | -9 | | 32 | Precentral & postcentral gyri | |
| 41 | 25.7 | | 13 | -39 | | 72 | Postcentral gyrus | |
| 40 | 21.2 | | -49 | 13 | | 18 | inferior frontal gyrus (p. triangularis/p. opercularis) | |
| ***Negative Parenting X Condition*** | | | | | | | | |
| **k** | **χ^2^(2)** | | **x** | **y** | | **z** | **Region** | |
| 45 | 25.8 | | 23 | 41 | | -4 | Superior orbital gyrus | |
| 42 | 22.5 | | -13 | -51 | | 4 | Calcarine & lingual gyri | |
| 41 | 23.0 | | -33 | 49 | | 12 | Middle frontal gyrus | |
| ***Negative Parenting X Accuracy*** | | | | | | | | |
| **k** | **χ^2^(2)** | **x** | | **y** | | **z** | | **Region** |
| 87 | 24.8 | 49 | | -5 | | 34 | | Precentral & postcentral gyri |
| 52 | 33.2 | 21 | | 5 | | 24 | | Caudate nucleus |
| 46 | 23.1 | 41 | | -15 | | -10 | | Hippocampus |
| ***Negative Parenting X Irritability*** | | | | | | | | |
| **k** | **χ^2^(2)** | **x** | | **y** | | **z** | | **Region** |
| 606 | 44.4 | -49 | | -13 | | 44 | | Postcentral & precentral gyri |
| 513 | 48.2 | | 5 | -69 | | 34 | Precuneus | |
| 498 | 36.6 | | 45 | -41 | | 34 | Inferior/superior parietal lobule,  supramarginal & angular gyri | |
| 276 | 46.1 | | 53 | -3 | | 34 | Precentral & postcentral gyri | |
| 180 | 30.3 | | 49 | 1 | | 42 | Precentral & middle frontal gyri | |
| 174 | 34.7 | | -1 | -35 | | 32 | Middle/posterior cingulate cortex | |
| 168 | 46.3 | | -37 | -55 | | 22 | Middle temporal & angular gyri | |
| 162 | 26.4 | | 37 | 57 | | 22 | Middle frontal & middle orbital gyri | |
| 138 | 29.9 | | 33 | -7 | | 14 | Putamen, insula lobe | |
| 116 | 32.6 | | 39 | -61 | | 10 | Middle temporal & middle occipital gyri | |
| 110 | 33.1 | | 51 | 23 | | 28 | middle frontal & inferior frontal (p. triangularis) gyri | |
| 101 | 51.1 | | 7 | -73 | | 8 | Calcarine & lingual gyri | |
| 95 | 28.2 | | -31 | -11 | | -6 | Putamen | |
| 82 | 25.6 | | 53 | -41 | | 4 | Middle temporal & superior temporal gyri | |
| 82 | 33.2 | | -45 | -55 | | 42 | Inferior parietal lobule, angular gyrus | |
| 69 | 44.4 | | 55 | -29 | | 22 | Superior temporal & supramarginal gyri, rolandic operculum | |
| 64 | 34.9 | | 57 | 7 | | 8 | Temporal pole, rolandic operculum,  inferior frontal gyrus (p. opercularis), | |
| 61 | 43.7 | | 61 | -45 | | 26 | Supramarginal & superior temporal gyri | |
| 57 | 30.2 | | 55 | -7 | | 16 | Rolandic operculum, postcentral gyrus | |
| 54 | 33.1 | | -13 | -15 | | 2 | Thalamus | |
| 47 | 28.9 | | 47 | 11 | | 16 | Inferior frontal gyrus (p. opercularis) & Superior medial gyri | |
| 44 | 31.2 | | 3 | 31 | | 36 | Middle cingulate cortex | |
| 42 | 26.0 | | 45 | 9 | | 32 | Inferior frontal gyrus (p. opercularis) | |
| ***Negative Parenting X Condition X Accuracy*** | | | | | | | | |
| **k** | **χ^2^(2)** | | **x** | **y** | | **Z** | **Region** | |
| 48 | 23.2 | | -59 | -27 | | 18 | Supramarginal, postcentral & superior temporal gyri | |
| 48 | 23.5 | | -9 | -41 | | 64 | Precuneus, paracentral lobule | |
| ***Negative Parenting X Irritability X Condition*** | | | | | | | | |
| **k** | **χ^2^(2)** | | **x** | **y** | **Z** | | **Region** | |
| 216 | 42.7 | | 51 | -5 | 32 | | Postcentral & precentral gyri | |
| 106 | 41.3 | | -43 | -13 | 28 | | Postcentral & precentral gyri | |
| 58 | 31.2 | | -45 | -13 | 42 | | Postcentral gyrus | |
| 48 | 32.8 | | -41 | -23 | -10 | | Middle temporal & postcentral gyri, | |
| 47 | 20.4 | | 59 | -11 | 18 | | rolandic operculum  Superior temporal gyrus | |
| 40 | 28.8 | | 41 | -61 | 52 | | Superior/inferior parietal lobule, angular gyrus, | |
| ***Negative Parenting X Irritability X Accuracy*** | | | | | | | | |
| **k** | **χ^2^(2)** | | **x** | **y** | **z** | | **Region** | |
| 865 | 50.8 | | -49 | -13 | 42 | | Postcentral & precentral gyri,  rolandic operculum | |
| 629 | 52.0 | | 47 | -5 | 30 | | Precentral & postcentral gyri | |
| 391 | 38.0 | | 45 | -41 | 34 | | Inferior/superior parietal lobule,  supramarginal gyrus | |
| 341 | 35.6 | | 5 | -71 | 34 | | Precuneus, cuneus | |
| 302 | 45.9 | | 57 | 9 | 6 | | Inferior Frontal Gyrus (p. Opercularis/p. Triangularis),  Rolandic Operculum,  Insula Lobe | |
| 206 | 46.2 | | 7 | -73 | 8 | | Calcarine & lingual gyri | |
| 170 | 40.3 | | 1 | -29 | 26 | | Posterior/middle cingulate cortex | |
| 158 | 44.9 | | -37 | -55 | 22 | | Middle temporal & angular gyri | |
| 144 | 27.4 | | 37 | 41 | 10 | | Middle frontal, middle orbital, & inferior  frontal (p. Orbitalis) gyri | |
| 135 | 28.9 | | -23 | -7 | 4 | | Insula lobe, putamen | |
| 129 | 32.0 | | 27 | -9 | 4 | | Putamen, insula lobe | |
| 118 | 30.7 | | 63 | -45 | 8 | | Middle temporal & superior temporal gyri | |
| 118 | 34.0 | | 51 | 23 | 26 | | inferior frontal (p. Triangularis) & middle frontal gyri | |
| 107 | 29.0 | | 63 | -5 | 18 | | Rolandic operculum, postcentral gyrus | |
| 85 | 31.9 | | 11 | -19 | 4 | | Thalamus | |
| 85 | 37.4 | | 37 | -63 | 12 | | Middle temporal & middle occipital gyri | |
| 82 | 39.0 | | 5 | -27 | 52 | | Middle cingulate cortex, SMA | |
| 76 | 44.8 | | 55 | -29 | 22 | | Superior temporal & supramarginal gyri,  rolandic operculum | |
| 70 | 30.9 | | -11 | -67 | 24 | | Precuneus, cuneus, superior occipital gyrus | |
| 67 | 43.6 | | 61 | -45 | 26 | | Supramarginal & superior temporal gyri | |
| 66 | 28.6 | | -49 | -41 | 38 | | Inferior parietal lobule | |
| 58 | 31.7 | | -47 | -65 | 14 | | Middle temporal, angular & middle occipital gyri | |
| 56 | 25.7 | | -29 | 57 | -6 | | Middle orbital gyrus, | |
| 51 | 25.9 | | 1 | -3 | 54 | | SMA, middle cingulate cortex | |
| 50 | 21.4 | | 5 | -67 | -2 | | Lingual gyrus | |
| 45 | 27.2 | | 39 | -9 | 54 | | Middle/superior frontal & precentral gyri | |
| 44 | 24.8 | | 5 | 65 | -6 | | Mid orbital & rectal gyri | |
| ***Negative Parenting X Irritability X Condition X Accuracy*** | | | | | | | | |
| **k** | **χ^2^(2)** | | **x** | **y** | | **z** | **Region** | |
| 197 | 40.5 | | 51 | -5 | | 32 | Precentral & postcentral gyri | |
| 92 | 38.6 | | -43 | -13 | | 28 | Postcentral & precentral gyri | |
| 74 | 38.2 | | 7 | -71 | | 34 | Precuneus, cuneus | |
| 64 | 32.7 | | -17 | 39 | | 18 | Middle Frontal Gyrus | |
| 61 | 36.2 | | -41 | 23 | | -10 | Hippocampus, middle temporal gyrus | |
| 43 | 26.7 | | -49 | 13 | | 44 | Postcentral & precentral gyri | |

**Table S3. Model Results from Connectivity Analysis with Cluster Extent by Overlap**

| **R Amygdala Seed** | | | | |
| --- | --- | --- | --- | --- |
| ***Negative Parenting Main Effect*** | | | | |
| **Cluster** | **Size (k)** | **Overlap** | **Region** | |
| 1 | 63 | 55.2 % | R_Precentral_Gyrus | |
|  |  | 35.2% | R_Postcentral_Gyrus | |
| 2 | 41 | 88.1 % | R_Postcentral_Gyrus | |
| 3 | 40 | 53.5 % | L_Inferior_Frontal_Gyrus_(p._Triangularis) | |
|  |  | 46.5 % | L_Inferior_Frontal_Gyrus_(p._Opercularis) | |
| ***Negative Parenting X Condition*** | | | | |
| **Cluster** | **Size (k)** | **Overlap** | **Region** | |
| 1 | 45 | 13.9 % | R_Superior_Orbital_Gyrus | |
| 2 | 42 | 68.5 % | L_Calcarine_Gyrus | |
|  |  | 28.8 % | L_Lingual_Gyrus | |
| 3 | 41 | 93.9 % | L_Middle_Frontal_Gyrus | |
| ***Negative Parenting X Accuracy*** | | | | |
| **Cluster** | **Size (k)** | **Overlap** | **Region** | |
| 1 | 87 | 58.4 % | R_Precentral_Gyrus | |
|  |  | 37.1 % | R_Postcentral_Gyrus | |
| 2 | 52 | 13.9 % | R_Caudate_Nucleus, | |
| 3 | 46 | 15.6 % | R_Hippocampus | |
| ***Negative Parenting X Irritability*** | | | | |
| **Cluster** | **Size (k)** | **Overlap** | | **Region** |
| 1 | 606 | 49.0 % | | L_Postcentral_Gyrus |
|  |  | 21.6 % | | L_Precentral_Gyrus |
|  |  | 17.6 % | | L_Rolandic_Operculum |
| 2 | 513 | 43.6 % | | L_Precuneus |
|  |  | 40.5 % | | R_Precuneus |
| 3 | 498 | 44.8 % | | R_Inferior_Parietal_Lobule |
|  |  | 27.8 % | | R_Superior_Parietal_Lobule |
|  |  | 13.6 % | | R_SupraMarginal_Gyrus |
|  |  | 11.6 % | | R_Angular_Gyrus |
| 4 | 276 | 60.3 % | | R_Precentral_Gyrus |
|  |  | 28.9 % | | R_Postcentral_Gyrus |
| 5 | 180 | 60.1 % | | R_Precentral_Gyrus |
|  |  | 26.7 % | | R_Middle_Frontal_Gyrus |
| 6 | 174 | 30.7 % | | R_Middle_Cingulate_Cortex |
|  |  | 22.5 % | | L_Posterior_Cingulate_Cortex |
|  |  | 13.6 % | | L_Middle_Cingulate_Cortex |
| 7 | 168 | 43.6 % | | L_Middle_Temporal_Gyrus |
|  |  | 43.5 % | | L_Angular_Gyrus |
| 8 | 162 | 51.3 % | | R_Middle_Frontal_Gyrus |
|  |  | 17.6 % | | R_Middle_Orbital_Gyrus, |
| 9 | 138 | 56.9 % | | R_Putamen |
|  |  | 13.5 % | | R_Insula_Lobe |
| 10 | 116 | 38.2 % | | R_Middle_Temporal_Gyrus |
|  |  | 25.2 % | | R_Middle_Occipital_Gyrus |
| 11 | 110 | 52.5 % | | R_Middle_Frontal_Gyrus, |
|  |  | 46.6 % | | R_Inferior_Frontal_Gyrus_(p._Triangularis) |
| 12 | 101 | 70.8 % | | R_Calcarine_Gyrus |
|  |  | 23.2 % | | R_Lingual_Gyrus |
| 13 | 95 | 46.2 % | | L_Putamen |
| 14 | 82 | 77.0 % | | R_Middle_Temporal_Gyrus |
|  |  | 21.8 % | | R_Superior_Temporal_Gyrus |
| 15 | 82 | 79.1 % | | L_Inferior_Parietal_Lobule |
|  |  | 14.7 % | | L_Angular_Gyrus |
| 16 | 69 | 46.5 % | | R_Superior_Temporal_Gyrus |
|  |  | 39.5 % | | R_SupraMarginal_Gyrus |
|  |  | 14.0 % | | R_Rolandic_Operculum |
| 17 | 64 | 34.5 % | | R_Temporal_Pole |
|  |  | 32.8 % | | R_Rolandic_Operculum, |
|  |  | 30.5 % | | R_Inferior_Frontal_Gyrus_(p._Opercularis) |
| 18 | 61 | 76.2 % | | R_SupraMarginal_Gyrus |
|  |  | 11.7 % | | R_Superior_Temporal_Gyrus |
| 19 | 57 | 75.8 % | | R_Rolandic_Operculum |
|  |  | 17.2 % | | R_Postcentral_Gyrus |
| 20 | 54 | 61.2 % | | L_Thalamus |
|  |  | 15.6 % | | R_Thalamus |
| 21 | 47 | 90.1 % | | R_Inferior_Frontal_Gyrus_(p._Opercularis) |
| 22 | 44 | 38.4 % | | R_Middle_Cingulate_Cortex |
|  |  | 32.5 % | | L_Superior_Medial_Gyrus |
|  |  | 28.7 % | | R_Superior_Medial_Gyrus |
| 23 | 42 | 91.4 % | | R_Inferior_Frontal_Gyrus_(p._Opercularis) |
| ***Negative Parenting X Condition X Accuracy*** | | | | |
| **Cluster** | **Size (k)** | **Overlap** | | **Region** |
| 1 | 48 | 29.7 % | | L_SupraMarginal_Gyrus |
|  |  | 26.2 % | | L_Postcentral_Gyrus |
|  |  | 18.4 % | | L_Superior_Temporal_Gyrus |
| 2 | 48 | 68.8 % | | L_Precuneus |
|  |  | 23.9 % | | L_Paracentral_Lobule |
| ***Negative Parenting X Irritability X Condition*** | | | | |
| **Cluster** | **Size (k)** | **Overlap** | | **Region** |
| 1 | 216 | 60.8 % | | R_Precentral_Gyrus |
|  |  | 32.0 % | | R_Postcentral_Gyrus |
| 2 | 106 | 65.9 % | | L_Postcentral_Gyrus |
|  |  | 25.2 % | | L_Precentral_Gyrus |
| 3 | 58 | 86.0 % | | L_Postcentral_Gyrus |
| 4 | 48 | 15.0 % | | L_Middle_Temporal_Gyrus |
| 5 | 47 | 73.1 % | | R_Rolandic_Operculum |
|  |  | 11.9 % | | R_Postcentral_Gyrus |
|  |  | 11.7 % | | R_Superior_Temporal_Gyrus |
| 6 | 40 | 59.0 % | | R_Superior_Parietal_Lobule |
|  |  | 19.6 % | | R_Inferior_Parietal_Lobule |
|  |  | 15.6 % | | R_Angular_Gyrus |
| ***Negative Parenting X Irritability X Accuracy*** | | | | |
| **Cluster** | **Size (k)** | **Overlap** | | **Region** |
| 1 | 865 | 43.2 % | | L_Postcentral_Gyrus, |
|  |  | 23.4 % | | L_Precentral_Gyrus |
|  |  | 13.5 % | | L_Rolandic_Operculum |
| 2 | 629 | 57.9 % | | R_Precentral_Gyrus |
|  |  | 23.5 % | | R_Postcentral_Gyrus |
| 3 | 391 | 47.1 % | | R_Inferior_Parietal_Lobule |
|  |  | 27.7 % | | R_Superior_Parietal_Lobule |
|  |  | 17.6 % | | R_SupraMarginal_Gyrus |
| 4 | 341 | 44.9 % | | R_Precuneus |
|  |  | 35.5 % | | L_Precuneus |
|  |  | 11.6 % | | R_Cuneus |
| 5 | 302 | 33.8 % | | R_Inferior_Frontal_Gyrus_(p._Opercularis) |
|  |  | 17.6 % | | R_Rolandic_Operculum |
|  |  | 13.7 % | | R_Insula_Lobe |
|  |  | 10.8 % | | R_Inferior_Frontal_Gyrus_(p._Triangularis) |
| 6 | 206 | 73.6 % | | R_Calcarine_Gyrus |
|  |  | 22.8 % | | R_Lingual_Gyrus |
| 7 | 170 | 19.8 % | | L_Posterior_Cingulate_Cortex |
|  |  | 19.7 % | | R_Middle_Cingulate_Cortex |
|  |  | 15.7 % | | L_Middle_Cingulate_Cortex |
| 8 | 158 | 50.2 % | | L_Middle_Temporal_Gyrus |
|  |  | 33.5 % | | L_Angular_Gyrus |
| 9 | 144 | 35.4 % | | R_Middle_Frontal_Gyrus |
|  |  | 25.3 % | | R_Middle_Orbital_Gyrus |
|  |  | 11.0 % | | R_Inferior_Frontal_Gyrus_(p._Orbitalis) |
| 10 | 135 | 39.9 % | | L_Insula_Lobe |
|  |  | 32.7 % | | L_Putamen |
| 11 | 129 | 55.9 % | | R_Putamen |
|  |  | 13.1 % | | R_Insula_Lobe |
| 12 | 118 | 80.7 % | | R_Middle_Temporal_Gyrus |
|  |  | 19.0 % | | R_Superior_Temporal_Gyrus |
| 13 | 118 | 57.5 % | | R_Inferior_Frontal_Gyrus_(p._Triangularis) |
|  |  | 41.0 % | | R_Middle_Frontal_Gyrus |
| 14 | 107 | 58.6 % | | R_Rolandic_Operculum |
|  |  | 29.6 % | | R_Postcentral_Gyrus |
| 15 | 85 | 61.4 % | | L_Thalamus |
|  |  | 17.8 % | | R_Thalamus |
| 16 | 85 | 29.8 % | | R_Middle_Temporal_Gyrus |
|  |  | 29.6 % | | R_Middle_Occipital_Gyrus |
| 17 | 82 | 48.5 % | | R_Middle_Cingulate_Cortex |
|  |  | 41.9 % | | R_SMA |
| 18 | 76 | 48.3 % | | R_Superior_Temporal_Gyrus |
|  |  | 40.5 % | | R_SupraMarginal_Gyrus |
|  |  | 11.2 % | | R_Rolandic_Operculum |
| 19 | 70 | 38.6 % | | L_Precuneus |
|  |  | 30.2 % | | L_Cuneus |
|  |  | 25.3 % | | L_Superior_Occipital_Gyrus |
| 20 | 67 | 77.0 % | | R_SupraMarginal_Gyrus |
|  |  | 11.8 % | | R_Superior_Temporal_Gyrus |
| 21 | 66 | 96.3 % | | L_Inferior_Parietal_Lobule |
| 22 | 58 | 53.9 % | | L_Middle_Temporal_Gyrus |
|  |  | 26.1 % | | L_Angular_Gyrus |
|  |  | 16.8 % | | L_Middle_Occipital_Gyrus |
| 23 | 56 | 73.8 % | | L_Middle_Orbital_Gyrus, |
| 24 | 51 | 60.0 % | | L_SMA |
|  |  | 23.8 % | | R_SMA |
|  |  | 16.2 % | | L_Middle_Cingulate_Cortex |
| 25 | 50 | 85.9 % | | R_Lingual_Gyrus |
| 26 | 45 | 57.0 % | | R_Middle_Frontal_Gyrus |
|  |  | 23.5 % | | R_Precentral_Gyrus |
|  |  | 19.5 % | | R_Superior_Frontal_Gyrus |
| 27 | 44 | 13.9 % | | R_Mid_Orbital_Gyrus |
|  |  | 10.3 % | | R_Rectal_Gyrus |
| ***Negative Parenting X Irritability X Condition X Accuracy*** | | | | |
| **Cluster** | **Size (k)** | **Overlap** | | **Region** |
| 1 | 197 | 57.3 % | | R_Precentral_Gyrus |
|  |  | 35.8 % | | R_Postcentral_Gyrus |
| 2 | 92 | 64.0 % | | L_Postcentral_Gyrus |
|  |  | 30.6 % | | L_Precentral_Gyrus, |
| 3 | 74 | 71.4 % | | R_Precuneus |
|  |  | 26.3 % | | R_Cuneus |
| 4 | 64 | 16.5 % | | L_Middle_Frontal_Gyrus |
| 5 | 61 | 29.1 % | | L_Hippocampus |
|  |  | 11.9 % | | L_Middle_Temporal_Gyrus |
| 6 | 43 | 87.8 % | | L_Postcentral_Gyrus |
|  |  | 11.2 % | | L_Precentral_Gyrus |

**Table S4. Model Results from Connectivity Analysis**

Significant cluster results show the associations between negative parenting scores, irritability symptoms, and neural connectivity during go/no-go conditions in the Whack-a-Mole task.

| **Right Amygdala Seed** | | | | | | |
| --- | --- | --- | --- | --- | --- | --- |
| ***Negative Parenting x Irritability*** | | | | | | |
| **k** | **χ^2^(2)** \| **x** \| **y** \| **z** \| **Region** | **x** | **y** | | **z** | **Region** |
| 56 | 26.7 | 5 | 65 | | -6 | Middle orbital gyrus |
| 47 | 23.8 | -51 | 25 | | -6 | Temporal Pole |
| 40 | 22.6 | -17 | 51 | | -8 | Middle/superior orbital gyri |
| 40 | 21.7 | 45 | -41 | | 32 | Supramarginal gyrus |
| ***Negative Parenting x Irritability x Condition*** | | | | | | |
| **k** | **χ^2^(2)** | **x** | **y** | **z** | | **Region** |
| 140 | 22.3 | -17 | 63 | 6 | | Superior medial & middle/superior orbital gyri |
| 84 | 28.4 | -55 | -31 | -10 | | Middle temporal gyrus |
| 82 | 25.2 | -43 | -19 | -6 | | Middle temporal gyrus |
| 72 | 18.3 | -3 | 27 | 16 | | Anterior cingulate cortex |
| 63 | 29.4 | -23 | 53 | -2 | | Superior/middle orbital gyri |
| 50 | 26.6 | 9 | 63 | 14 | | Superior medial & superior frontal gyrus |
| 49 | 19.9 | -53 | -49 | 14 | | Middle/superior temporal gyri |
| 46 | 19.1 | -41 | 17 | 24 | | Inferior frontal gyrus |
| 44 | 20.8 | 5 | 33 | 26 | | Anterior cingulate cortex |
| 43 | 21.9 | -41 | -29 | -10 | | Inferior frontal gyrus |
| 43 | 26.9 | -17 | 45 | 26 | | Superior/middle frontal gyri |
| 41 | 18.8 | -3 | -21 | 34 | | Middle cingulate cortex |
| 40 | 19.3 | -1 | 41 | 6 | | Anterior cingulate cortex |
| ***Negative Parenting x Irritability x Accuracy*** | | | | | | |
| **k** | **χ^2^(2)** | **x** | **y** | **z** | | **Region** |
| 49 | 22.9 | -25 | 33 | 42 | | Superior/middle frontal gyri |
| 48 | 24.2 | 51 | 29 | -8 | | Inferior frontal gyrus |
| 46 | 23.7 | -57 | 7 | 22 | | Inferior frontal gyrus |
| ***Negative Parenting x Irritability x Condition x Accuracy*** | | | | | | |
| **k** | **χ^2^(2)** | **x** | **y** | | **z** | **Region** |
| 106 | 20.7 | 5 | 35 | | 18 | Anterior cingulate gyrus |
| 65 | 24.9 | -41 | 29 | | -10 | Inferior frontal gyrus |
| 49 | 23.7 | -41 | -21 | | -8 | Middle temporal gyrus |
| 46 | 27.8 | 9 | -53 | | 66 | Precuneus, superior parietal lobule, postcentral gyrus |

**Table S5. Post-Hoc Analysis Model Results Across Contrasts of Interest**

| **Negative Parenting X Irritability** |  |  |
| --- | --- | --- |
| **Region** | **Covariate** | **Significance** |
| Postcentral & precentral gyri, rolandic operculum |  | *p* < .001 |
|  | Sex | *p* = .003 |
|  | SES | *p* = .004 |
|  | Race/Ethnicity | *p* = .003 |
|  | Average Censored Motion | *p* < .001 |
| Precuneus |  | *p* = .009 |
|  | Sex | *p* = .008 |
|  | SES | *p* = .008 |
|  | Race/Ethnicity | *p* = .007 |
|  | Average Censored Motion | *p* = .004 |
| Inferior/superior parietal lobule,  supramarginal & angular gyri |  | *p* = .002 |
|  | Sex | *p* = .005 |
|  | SES | *p* = .004 |
|  | Race/Ethnicity | *p* = .008 |
|  | Average Censored Motion | *p* = .002 |
| Precentral & postcentral gyri |  | *p* < .001 |
|  | Sex | *p* = .005 |
|  | SES | *p* = .005 |
|  | Race/Ethnicity | *p* = .002 |
|  | Average Censored Motion | *p* < .001 |
| Precentral & middle frontal gyri |  | *p* = .003 |
|  | Sex | *p* = .008 |
|  | SES | *p* = .002 |
|  | Race/Ethnicity | *p* = .008 |
|  | Average Censored Motion | *p* = .003 |
| Middle/posterior cingulate cortex |  | *p* = .003 |
|  | Sex | *p* = .008 |
|  | SES | *p* = .006 |
|  | Race/Ethnicity | *p* = .007 |
|  | Average Censored Motion | *p* = .002 |
| Middle temporal & angular gyri |  | *p* = .006 |
|  | Sex | *p* = .007 |
|  | SES | *p* = .005 |
|  | Race/Ethnicity | *p* = .007 |
|  | Average Censored Motion | *p* = .004 |
| Middle frontal & middle orbital gyri |  | *p* < .001 |
|  | Sex | *p* = .002 |
|  | SES | *p* = .003 |
|  | Race/Ethnicity | *p* = .002 |
|  | Average Censored Motion | *p* < .001 |
| Middle temporal & middle occipital gyri |  | *p* = .007 |
|  | Sex | *p* = .007 |
|  | SES | *p* = .009 |
|  | Race/Ethnicity | *p* = .007 |
|  | Average Censored Motion | *p* = .007 |
| Middle frontal & inferior frontal  (p. triangularis) gyri |  | *p* < .001 |
|  | Sex | *p* = .003 |
|  | SES | *p* = .007 |
|  | Race/Ethnicity | *p* = .008 |
|  | Average Censored Motion | *p* < .001 |
| Middle temporal & superior temporal gyri |  | *p* = .009 |
|  | Sex | *p* = .009 |
|  | SES | *p* = .007 |
|  | Race/Ethnicity | *p* = .009 |
|  | Average Censored Motion | *p* = .003 |
| Inferior parietal lobule, angular gyrus |  | *p* < .001 |
|  | Sex | *p* = .008 |
|  | SES | *p* = .008 |
|  | Race/Ethnicity | *p* = .006 |
|  | Average Censored Motion | *p* = .004 |
| Superior temporal & supramarginal gyri, rolandic operculum |  | *p* = .007 |
|  | Sex | *p* = .006 |
|  | SES | *p* = .006 |
|  | Race/Ethnicity | *p* = .007 |
|  | Average Censored Motion | *p* = .005 |
| Temporal pole, rolandic operculum, inferior frontal gyrus (p. opercularis) |  | *p* = .009 |
|  | Sex | *p* = .007 |
|  | SES | *p* = .008 |
|  | Race/Ethnicity | *p* = .007 |
|  | Average Censored Motion | *p* = .002 |
| Supramarginal & superior temporal gyri |  | *p* = .008 |
|  | Sex | *p* = .004 |
|  | SES | *p* = .006 |
|  | Race/Ethnicity | *p* = .006 |
|  | Average Censored Motion | *p* = .002 |
| Rolandic operculum, postcentral gyrus |  | *p* < .001 |
|  | Sex | *p* = .004 |
|  | SES | *p* = .005 |
|  | Race/Ethnicity | *p* = .002 |
|  | Average Censored Motion | *p* < .001 |
| Inferior frontal gyrus (p. opercularis) & superior medial gyri |  | *p* < .001 |
|  | Sex | *p* = .008 |
|  | SES | *p* = .007 |
|  | Race/Ethnicity | *p* = .005 |
|  | Average Censored Motion | *p* < .001 |
| Middle cingulate cortex |  | *p* < .001 |
|  | Sex | *p* = .002 |
|  | SES | *p* = .002 |
|  | Race/Ethnicity | *p* = .003 |
|  | Average Censored Motion | *p* = .002 |
| Inferior frontal gyrus (p. opercularis) |  | *p* < .001 |
|  | Sex | *p* = .003 |
|  | SES | *p* < .001 |
|  | Race/Ethnicity | *p* = .004 |
|  | Average Censored Motion | *p* < .001 |
| **Negative Parenting X Irritability X Condition** |  |  |
| **Region** | **Covariate** | **Significance** |
| Postcentral & precentral gyri |  | *p* < .001 |
|  | Sex | *p* = .009 |
|  | SES | *p* = .004 |
|  | Race/Ethnicity | *p* = .007 |
|  | Average Censored Motion | *p* < .001 |
| Postcentral & precentral gyri |  | *p* < .001 |
|  | Sex | *p* = .002 |
|  | SES | *p* = .004 |
|  | Race/Ethnicity | *p* = .003 |
|  | Average Censored Motion | *p* < .001 |
| Postcentral gyrus |  | *p* < .001 |
|  | Sex | *p* = .005 |
|  | SES | *p* < .001 |
|  | Race/Ethnicity | *p* = .004 |
|  | Average Censored Motion | *p* < .001 |
| Middle temporal & postcentral gyri |  | *p* < .001 |
|  | Sex | *p* = .009 |
|  | SES | *p* = .003 |
|  | Race/Ethnicity | *p* = .005 |
|  | Average Censored Motion | *p* < .001 |
| Rolandic operculum, superior temporal gyrus |  | *p* = .009 |
|  | Sex | *p* = .009 |
|  | SES | *p* = .008 |
|  | Race/Ethnicity | *p* = .008 |
|  | Average Censored Motion | *p* = .005 |
| Superior/inferior parietal lobule, angular gyrus |  | *p* = .003 |
|  | Sex | *p* = .009 |
|  | SES | *p* = .009 |
|  | Race/Ethnicity | *p* = .009 |
|  | Average Censored Motion | *p* = .004 |
| **Negative Parenting X Irritability X Accuracy** |  |  |
| **Region** | **Covariate** | **Significance** |
| Postcentral & precentral gyri, rolandic operculum |  | *p* < .001 |
|  | Sex | *p* = .005 |
|  | SES | *p* = .004 |
|  | Race/Ethnicity | *p* = .007 |
|  | Average Censored Motion | *p* < .001 |
| Precentral & postcentral gyri |  | *p* < .001 |
|  | Sex | *p* = .008 |
|  | SES | *p* = .005 |
|  | Race/Ethnicity | *p* = .009 |
|  | Average Censored Motion | *p* < .001 |
| Inferior/superior parietal lobule, supramarginal gyrus |  | *p* = .003 |
|  | Sex | *p* = .005 |
|  | SES | *p* = .002 |
|  | Race/Ethnicity | *p* = .009 |
|  | Average Censored Motion | *p* = .003 |
| Precuneus, cuneus |  | *p* = .006 |
|  | Sex | *p* = .006 |
|  | SES | *p* = .007 |
|  | Race/Ethnicity | *p* = .006 |
|  | Average Censored Motion | *p* = .002 |
| Inferior Frontal Gyrus (p. Opercularis/p. Triangularis), rolandic operculum, insula lobe |  | *p* < .001 |
|  | Sex | *p* = .005 |
|  | SES | *p* = .006 |
|  | Race/Ethnicity | *p* = .008 |
|  | Average Censored Motion | *p* < .001 |
| Calcarine & lingual gyri |  | *p* = .002 |
|  | Sex | *p* = .007 |
|  | SES | *p* = .006 |
|  | Race/Ethnicity | *p* = .006 |
|  | Average Censored Motion | *p* < .001 |
| Posterior/middle cingulate cortex |  | *p* < .001 |
|  | Sex | *p* = .005 |
|  | SES | *p* = .002 |
|  | Race/Ethnicity | *p* = .005 |
|  | Average Censored Motion | *p* < .001 |
| Middle temporal & angular gyri |  | *p* = .007 |
|  | Sex | *p* = .008 |
|  | SES | *p* = .007 |
|  | Race/Ethnicity | *p* = .006 |
|  | Average Censored Motion | *p* = .003 |
| Middle frontal, middle orbital, & inferior frontal (p. Orbitalis) gyri |  | *p* < .001 |
|  | Sex | *p* = .005 |
|  | SES | *p* = .002 |
|  | Race/Ethnicity | *p* = .002 |
|  | Average Censored Motion | *p* < .001 |
| Insula lobe, putamen |  | *p* = .006 |
|  | Sex | *p* = .007 |
|  | SES | *p* = .004 |
|  | Race/Ethnicity | *p* = .008 |
|  | Average Censored Motion | *p* = .003 |
| Putamen, insula lobe |  | *p* = .007 |
|  | Sex | *p* = .009 |
|  | SES | *p* = .008 |
|  | Race/Ethnicity | *p* = .008 |
|  | Average Censored Motion | *p* = .004 |
| Middle temporal & superior temporal gyri |  | *p* = .005 |
|  | Sex | *p* = .006 |
|  | SES | *p* = .004 |
|  | Race/Ethnicity | *p* = .006 |
|  | Average Censored Motion | *p* = .002 |
| Inferior frontal (p. Triangularis) & middle frontal gyri |  | *p* < .001 |
|  | Sex | *p* = .005 |
|  | SES | *p* = .004 |
|  | Race/Ethnicity | *p* = .005 |
|  | Average Censored Motion | *p* < .001 |
| Rolandic operculum, postcentral gyrus |  | *p* = .002 |
|  | Sex | *p* = .009 |
|  | SES | *p* = .008 |
|  | Race/Ethnicity | *p* = .009 |
|  | Average Censored Motion | *p* = .002 |
| Thalamus |  | *p* < .001 |
|  | Sex | *p* = .004 |
|  | SES | *p* = .002 |
|  | Race/Ethnicity | *p* = .005 |
|  | Average Censored Motion | *p* < .001 |
| Middle temporal & middle occipital gyri |  | *p* = .006 |
|  | Sex | *p* = .009 |
|  | SES | *p* = .006 |
|  | Race/Ethnicity | *p* = .007 |
|  | Average Censored Motion | *p* = .004 |
| Middle cingulate cortex, SMA |  | *p* < .001 |
|  | Sex | *p* = .004 |
|  | SES | *p* = .005 |
|  | Race/Ethnicity | *p* = .007 |
|  | Average Censored Motion | *p* < .001 |
| Superior temporal & supramarginal gyri, rolandic operculum |  | *p* = .005 |
|  | Sex | *p* = .008 |
|  | SES | *p* = .002 |
|  | Race/Ethnicity | *p* = .006 |
|  | Average Censored Motion | *p* = .002 |
| Precuneus, cuneus, superior occipital gyrus |  | *p* = .009 |
|  | Sex | *p* = .009 |
|  | SES | *p* = .004 |
|  | Race/Ethnicity | *p* = .008 |
|  | Average Censored Motion | *p* < .001 |
| Supramarginal & superior temporal gyri |  | *p* = .007 |
|  | Sex | *p* = .008 |
|  | SES | *p* = .006 |
|  | Race/Ethnicity | *p* = .008 |
|  | Average Censored Motion | *p* = .003 |
| Inferior parietal lobule |  | *p* = .003 |
|  | Sex | *p* = .009 |
|  | SES | *p* = .009 |
|  | Race/Ethnicity | *p* = .008 |
|  | Average Censored Motion | *p* = .002 |
| Middle temporal, angular & middle occipital gyri |  | *p* < .001 |
|  | Sex | *p* = .006 |
|  | SES | *p* = .005 |
|  | Race/Ethnicity | *p* = .007 |
|  | Average Censored Motion | *p* < .001 |
| Middle orbital gyrus |  | *p* = .003 |
|  | Sex | *p* = .008 |
|  | SES | *p* = .005 |
|  | Race/Ethnicity | *p* = .009 |
|  | Average Censored Motion | *p* = .003 |
| SMA, middle cingulate cortex |  | *p* = .002 |
|  | Sex | *p* = .004 |
|  | SES | *p* = .004 |
|  | Race/Ethnicity | *p* = .006 |
|  | Average Censored Motion | *p* = .003 |
| Middle/superior frontal & precentral gyri |  | *p* < .001 |
|  | Sex | *p* = .007 |
|  | SES | *p* = .008 |
|  | Race/Ethnicity | *p* = .007 |
|  | Average Censored Motion | *p* < .001 |
| Mid orbital & rectal gyri |  | *p* = .009 |
|  | Sex | *p* = .009 |
|  | SES | *p* = .004 |
|  | Race/Ethnicity | *p* = .008 |
|  | Average Censored Motion | *p* = .002 |
| **Negative Parenting X Irritability X Condition X Accuracy** | |  |
| **Region** | **Covariate** | **Significance** |
| Precentral & postcentral gyri |  | *p* < .001 |
|  | Sex | *p* = .007 |
|  | SES | *p* = .003 |
|  | Race/Ethnicity | *p* = .006 |
|  | Average Censored Motion | *p* < .001 |
| Postcentral & precentral gyri |  | *p* < .001 |
|  | Sex | *p* = .008 |
|  | SES | *p* < .001 |
|  | Race/Ethnicity | *p* = .005 |
|  | Average Censored Motion | *p* < .001 |
| Precuneus, cuneus |  | *p* < .001 |
|  | Sex | *p* = .007 |
|  | SES | *p* = .003 |
|  | Race/Ethnicity | *p* = .005 |
|  | Average Censored Motion | *p* < .001 |
| Middle frontal gyrus |  | *p* < .001 |
|  | Sex | *p* = .004 |
|  | SES | *p* = .002 |
|  | Race/Ethnicity | *p* = .006 |
|  | Average Censored Motion | *p* < .001 |
| Hippocampus, middle temporal gyrus |  | *p* = .009 |
|  | Sex | *p* = .009 |
|  | SES | *p* = .006 |
|  | Race/Ethnicity | *p* = .009 |
|  | Average Censored Motion | *p* = .005 |
| Postcentral & precentral gyri |  | *p* = .002 |
|  | Sex | *p* = .005 |
|  | SES | *p* = .004 |
|  | Race/Ethnicity | *p* = .008 |
|  | Average Censored Motion | *p* < .001 |
